# Supplementary material for: Dynamic FRET-FLIM based screening of signal transduction pathways
Source: Sci Rep. 2021 Oct 20;11:20711. doi: 10.1038/s41598-021-00098-9 (PMC8528867; doi:10.1038/s41598-021-00098-9)

Supplementary material

Dynamic FRET-FLIM based screening of signal transduction pathways

Rolf Harkes[1,4], Olga Kukk[1,4], Sravasti Mukherjee[1,2], Jeffrey Klarenbeek[1], Bram van den Broek[1,3] & Kees Jalink[1,2]

1 Cell Biophysics Group, Department of Cell Biology, The Netherlands Cancer Institute, Amsterdam, The Netherlands.

2 Swammerdam Institute for Life Sciences, University of Amsterdam, Amsterdam, The Netherlands.

3 BioImaging Facility, The Netherlands Cancer Institute, Amsterdam, The Netherlands.

4 These authors contributed equally: Rolf Harkes and Olga Kukk.

Supplementary Table S1. siRNA oligonucleotides used for individual PDE knockdown. *Gene knockdown was achieved by transfection with a pool of four exogenous short RNA oligonucleotides 72 h prior to imaging.*

PDE1A	GCACUAAGACGAUCAAAUA	UAAAUGGUCUUUCGAUGUA	CAAAUAGGUUUCAUCGAUU	GGAAGCAGUUUAUAUCGAU
PDE1B	GCAAGAAGAUGUGGAUUAA	GGAAGUACAAGAAUCCUUA	GUUCAGUGCUGGAGAAUCA	GAUGAGACACGGCAAAUCU
PDE1C	CCAAGGAGAUUGAAGAAUU	GAUCAUGCACUGAAAUUUA	CAUCAUCGCUGGACAAUGU	GAACUACUCACACGUUAUG
PDE5A	GAAGACAGCUCCAAUGACA	GAAAUCAGGUGCUGCUUGA	GAUGACAGCUUGUGAUCUU	GGAAACGGUGGGACAUUUA
PDE2A	GAACAUCCCUGACGCAUAU	CCAAUGAGAUGAUGAUGUA	GAGCUGAUCUACAAAGAAU	CAACAUCUUUGAUCAUUUC
PDE3A	GAAGAUAUCCCGGUGUUUA	CAAGGUAAAUGAUGAUGUU	UGACACAACUGCCAAACAA	GAGAUUGGAUAUAGGGAUA
PDE3B	GAAUACAACUUCCUUCUUC	GACAUCAAAUGCUGAAAUA	GAACAGCAAACAAAUAUUG	GGUGAUAAGUGGCUAACAG
PDE4A	UCACACACCUGUCAGAAAU	CCAAGCCGCUGGAGCUGUA	GGACAACUGCGACAUCUUC	GUAACAGCCUGAACAACUC
PDE4B	GAAUGUAGCUUGAGUAAAU	GAACUUGCUUUGAUGUAUA	GGAACCAGGUGUCUGAAUA	GAAAGAGACCUCCUAAAGA
PDE4C	AGAGACAGCUUUAGCCAAA	CCUCACAGCUAUCAUAUUC	CCAACCAGUUUCUGAUUAA	GGGACGGGCCUGACAGAUU
PDE4D	GAAAUCAAGUGUCAGAGUU	GAACUUGCCUUGAUGUACA	CCAAGGAACUAGAAGAUGU	GAACGUGGCAUGGAGAUAA
PDE6D	UGACGACGAUCUUCUUGUA	CGUCUUAACUGGGAACGUU	CAAACUAAAUUGGAUGAAC	AUCCCUAACUCCACAAAUA
PDE7A	GCUAGGAGAUGUACGUGUA	GGAUAGAGGUGAUUUAUGC	GUACUUCCAUUUAGAUAUG	GCGUGGAGCUAUUUCCUAU
PDE7B	GAAAUCAGUCCUCUUUGUA	GGCGAAAUCUUGUUUGAGA	CAACAGGCAGAAUGAAUUU	GCUGGGAGAUAUACGACUA
PDE6G	GAAAGGCGUUCAAGGGUUU	GAACAGACAUCACAGUCAU	CGACAUCCCUGGAAUGGAA	CGACAGACCAGGCAGUUCA
PDE8A	GGAAAGGACUGGACGAAAU	CAAAGAAGAUAACCAAUGU	GAAGAUAACCAAUGUAAUG	GAAGUGAGCUGGCCAUUUU
PDE8B	GAGAAUAGCAGCAUAAUUG	GGUUAUAGAUGCAAUAUUG	GAAGUUCGCUCCCAGUUCA	GCGAUGACCACGUGAUUCA
PDE10A	GAACUAAACAGCUAUAUAG	GUAAUUGGUUUGAUGAUGA	GAACAAGGAGUUAUAUUCA	GCAGAGGCCUUGCCAAACA
PDE11A	GAAGAUUACUUGAUGCGGA	CCGACUGGCUAAUAAAUAA	UGAAGGAGCUCCAUUUACU	GGGAAGAGCUACACCAAAA
PDE6A	CAAGAGAGAUGAAGAGAUU	GAACAGGAGUGGACACAGU	CCAAUAACCUCUACCAGAU	GAGUCUGGAUGGAUGAUUA
PDE6B	GCACAGAAAUUUGCAAUGG	ACAAGGAGUUCUCUCGUUU	CAUUUGACAUCUACGAAUU	CCGGGUGGCUCAUCAAGAA
PDE6C	CAACUGACCUGGCUUUAUA	GAAAGGACCUGUAGACGAA	GGAUUUAUCUGUAACAUGA	CAGCAGAACUGUACGAAUU

Supplementary Table S2. Comparison of mean donor baseline lifetimes in unstimulated control wells. *Mean baseline values are calculated from all of segmented individual cells from the control wells of respective experimental day.*

	mean baseline ± SD, ns	number of cells	max. mean baseline value
Exp. day 20190502	2.30 ns ± 0.08	305	2.55
Exp. day 20191107	2.24 ns ± 0.04	1252	2.44
Exp. day 20200107	2.32 ns ± 0.04	135	2.49
Exp. day 20200206	2.29 ns ± 0.04	749	2.47

Supplementary Figure S1. Comparison of cell segmentation routines: Cellpose versus custom Voronoi-based Fiji routine. *Images show segmentation outcome (labelmaps, in which each cell ROI corresponds to a color label) overlayed on the intensity image. The Voronoi-based segmentation generates cell labels separated by a 1-pixel border, which in general appear somewhat more 'blocky' and occasionally include the cytosol (edges) of neighboring cells. The script is designed to remove very dim and/or very small cells. Cellpose produces more smooth labels that better follow the outlines of the cells, but for dim cells often produces labels that are too small. In our analysis, these cells are removed in later analysis steps.*

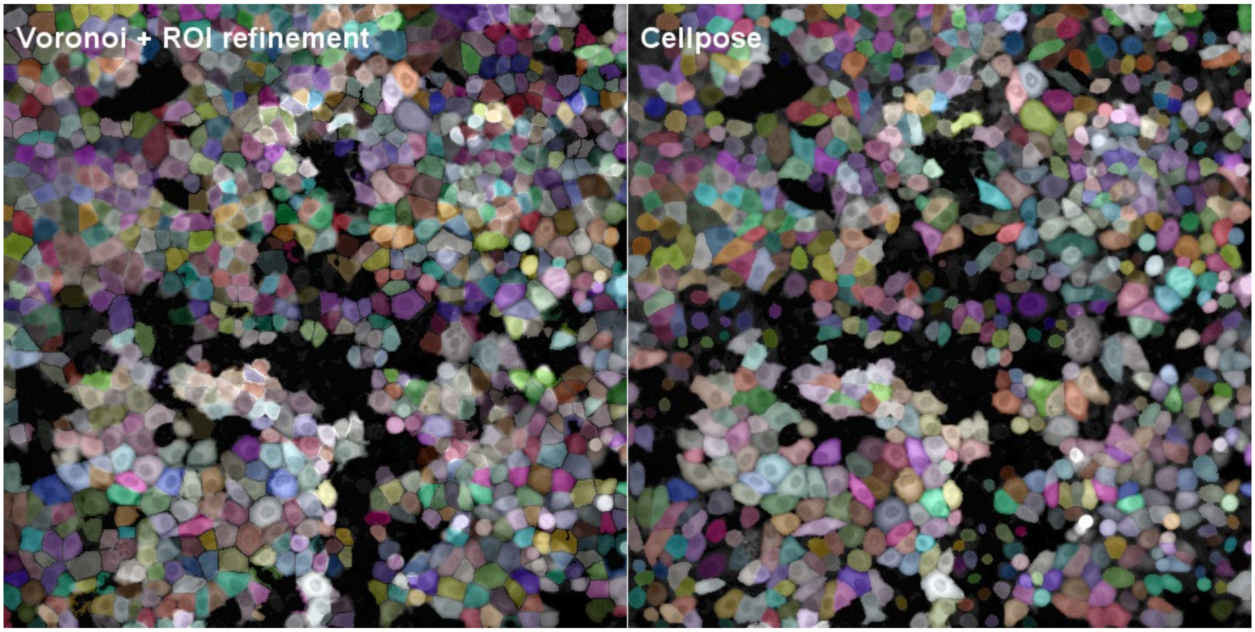

Supplementary Figure S2: Baseline lifetimes, and thus cAMP levels, are stable for the duration of the experiment. *Distribution of baseline lifetimes of all cells of a FOV in a well acquired at the onset of the screen (green, well B08), and a well acquired 6 hours later towards the end of the screen (blue, well G06). Well B08: 2.27 ns +/- 0.04 ns (mean +/- SD), max 2.44 ns, SEM = 0.00303, N = 210; well G06: 2.26 ns +/- 0.03 ns (mean +/- SD), max 2.39 ns, SEM = 0.00183, N=335).*

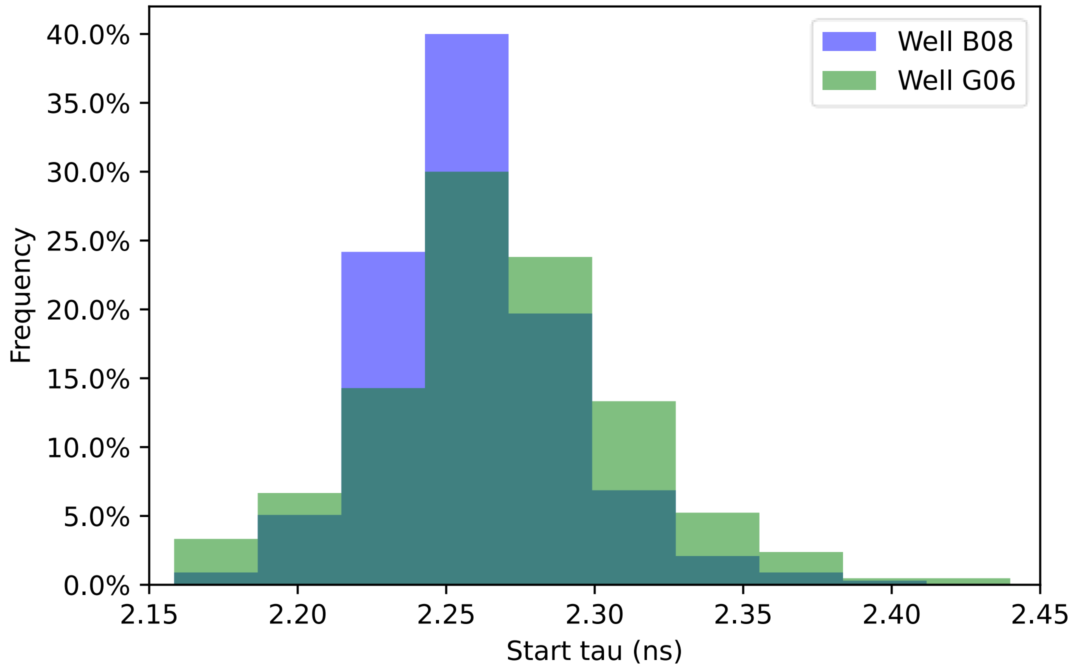

Supplementary Figure S3: Analysis of base cAMP levels in both uncaging and receptor-activation screens. *(A) Lifetime traces of all cells in an imaged FOV of a non-transfected control well in a DMNB-cAMP uncaging experiment and (B) in an experiment with GPCR-stimulation. Each trace represents the intensity-weighted mean of all pixels in one ROI (segmented cell). A clear raise in pre-stimulation baseline levels is detectable in cells loaded with DMNB-cAMP. (C, D) Plotting 5 selected traces taken from panels A and B visualizes that upon breakdown, cAMP levels typically return to their pre-stimulation values in both types of assays. (E, F) Plots of pre-stimulation values (average of 12 baseline samples) versus post-stimulation return values (average of last 8 samples in the trace) for all cells plotted in A and B. Note the clear correlation between return levels and baseline levels.*

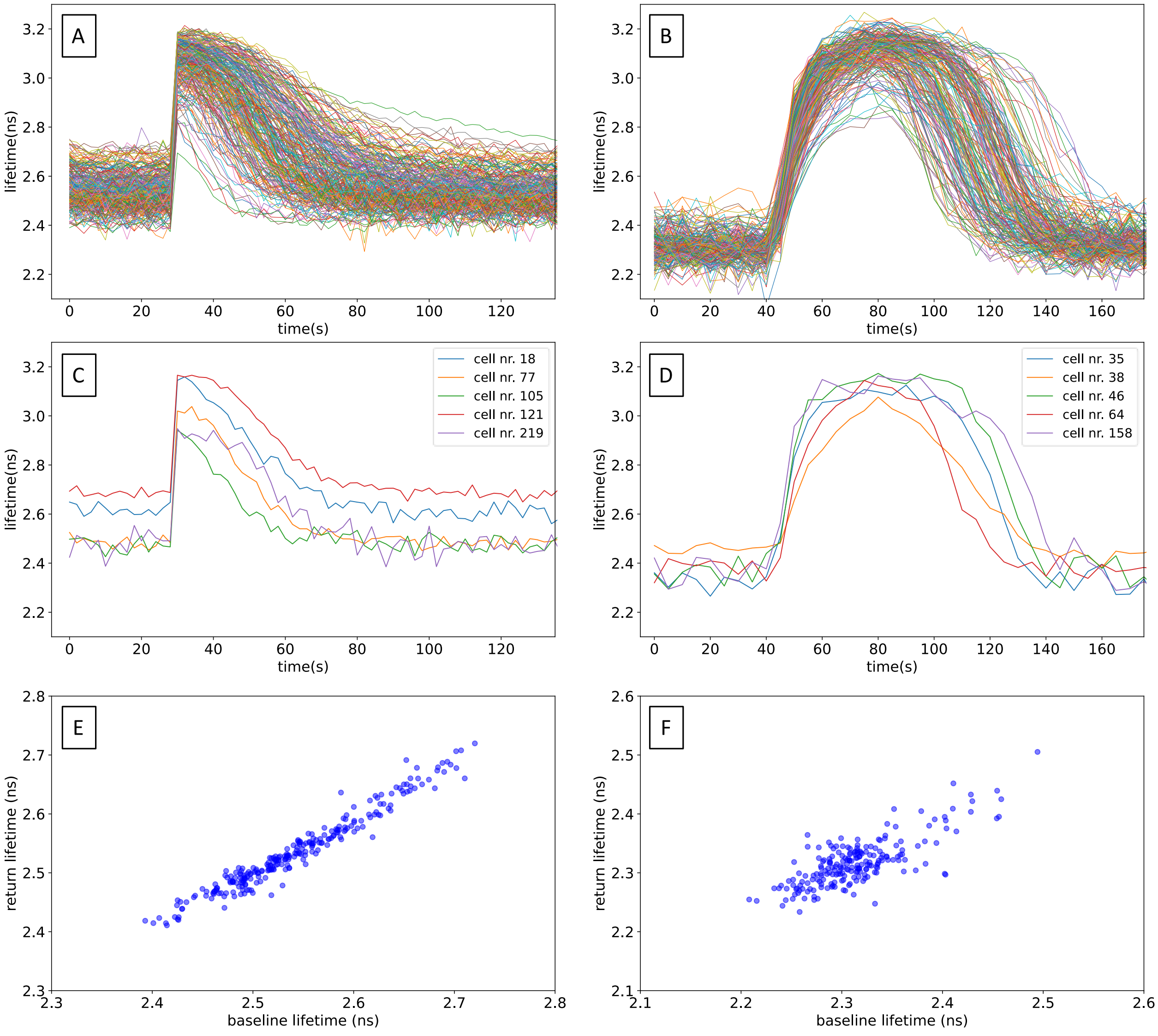

Supplementary Figure S4: PDE3A and PDE10A knockdown slightly affects baseline cAMP levels in uncaging screens, but not in GPCR stimulation screens. *(A) Initial (baseline) donor lifetime values for all ROIs in the two screens with DMNB-cAMP uncaging and (B) in the two screens with GPCR stimulation (i.e., no loading with DMNB-cAMP). Note that PDE3A and PDE10A knockdown cells exhibit a slightly higher lifetime in cells pretreated with DMNB-cAMP, suggesting that those phosphodiesterases play a role in controlling baseline cAMP levels. In DMNB-cAMP loaded cells, treatment with PDE inhibitors also affects baseline lifetimes more prominently than in GPCR stimulation screens. (C, D) The difference between pre-stimulation lifetime values and the corresponding return lifetimes for all ROIs from two screens with DMNB-caged cAMP loading (C) and two screens without caged cAMP loading (D). Cells were treated with siRNAs for the indicated PDEs. Datapoints are fitted decay times of single cells. Each experiment was performed twice independently with duplicate wells for each condition (i.e., 4 wells per condition). Indicated are median value (vertical black line), mean value (green dotted line); boxes encompass middle 50% of values and whiskers represent 1.5 times the interquartile range.*

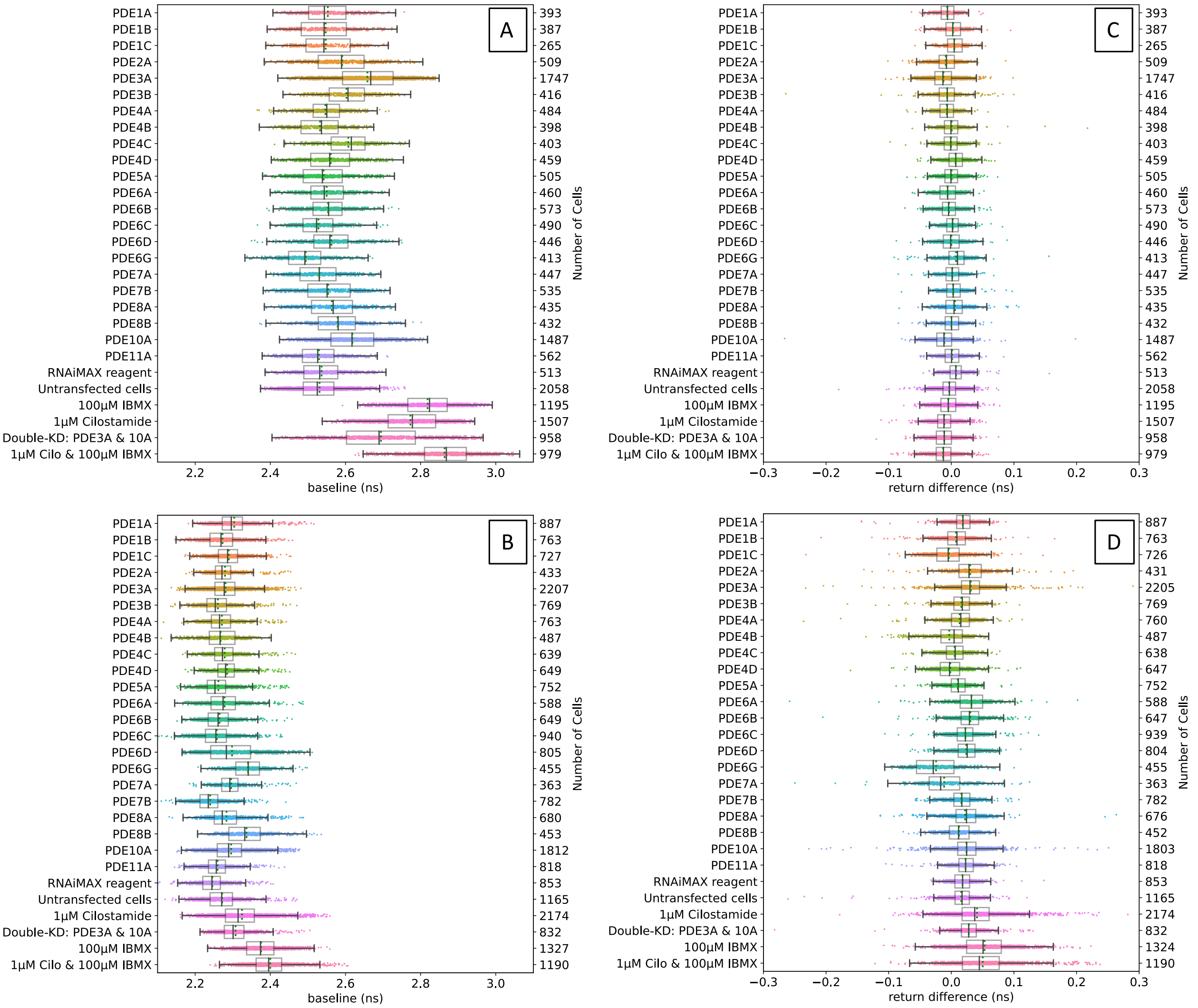

Supplementary Figure S5: Relationship between biosensor expression levels, baseline levels and cAMP breakdown rates. *Initial (baseline) donor lifetime values are plotted against the corresponding cAMP breakdown times for cells expressing above-average (green dots) or below-average (blue dots) levels of bio-sensors. Data are pooled from the duplicate control wells (A, D), the PDE3A-knockdown wells (B, E) and from wells pretreated with 1 µM cilostamide (C, F) from one experiment using DMNB-cAMP uncaging (A, B, C) and one involving GPCR stimulation (D, E, F) carried out on two different days.*

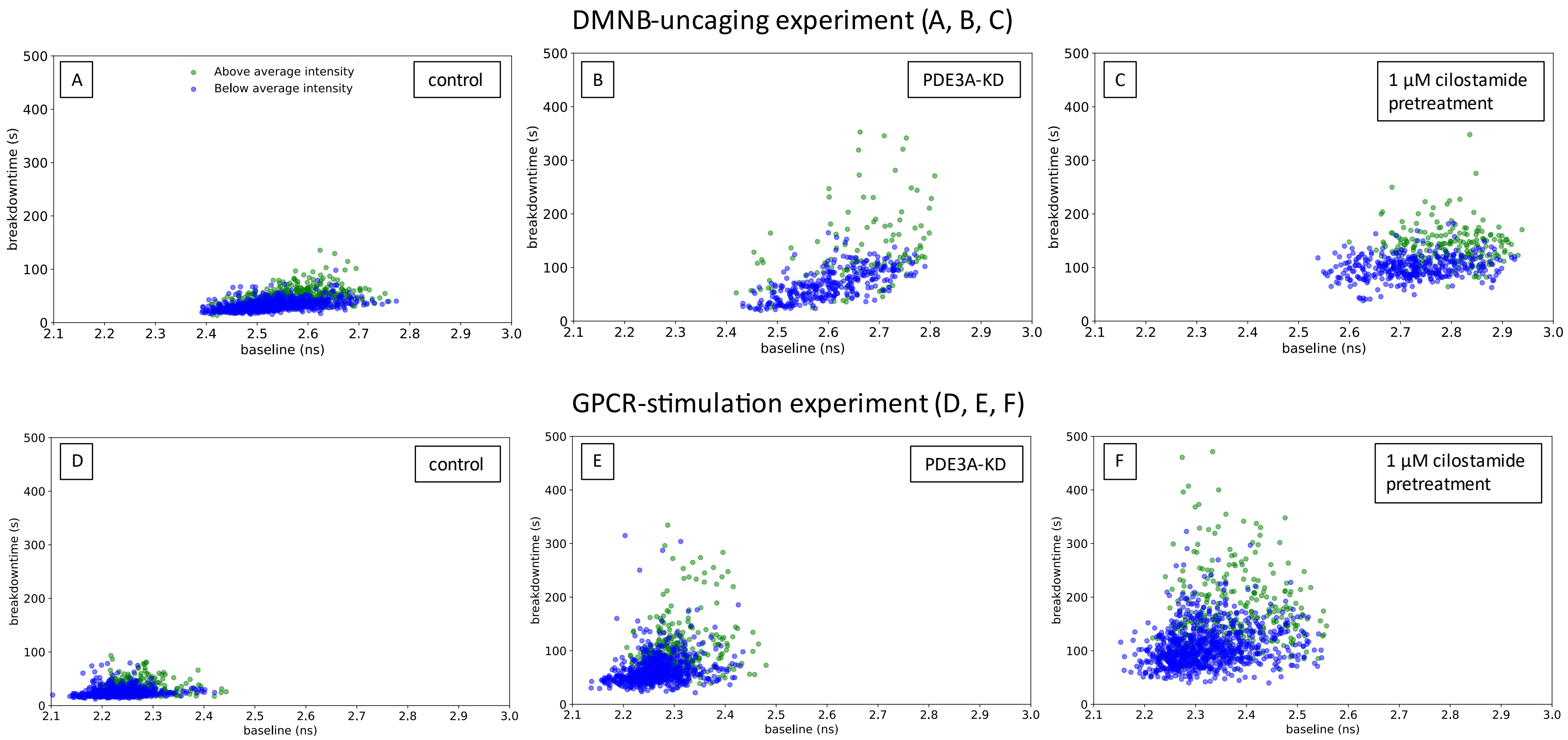

Supplement: Supplementary file 1 — Supplementary Information. [file 41598_2021_98_MOESM1_ESM.pdf]
